# Infantile Scurvy: Two Case Reports

**DOI:** 10.1155/2010/717518

**Published:** 2010-09-29

**Authors:** Leila Ghedira Besbes, Samir Haddad, Chebil Ben Meriem, Mondher Golli, Mohamed-fadhel Najjar, Mohamed-Néji Guediche

**Affiliations:** ^1^Pediatric Department, Fattouma Bourguiba Hospital, Monastir 5000, Tunisia; ^2^Radiology Department, Fattouma Bourguiba Hospital, Monastir 5000, Tunisia; ^3^Biochemistry Laboratory, Fattouma Bourguiba Hospital, Monastir 5000, Tunisia

## Abstract

*Background*. Ascorbic acid (vitamin C) is necessary for the formation of collagen, reducing free radicals, and aiding in iron absorption. SCURVY, a disease of dietary ascorbic acid deficiency, is uncommon today. It still exists in high risk groups including economically disadvantaged populations with poor nutrition. The incidence of SCURVY in the pediatric population is very low. *Cases Report*. Here we report two cases of SCURVY revealed by subperiosteal hematoma in children with cerebral palsy and developmental delay. *Conclusion*. SCURVY is extremely rare in children. Musculoskeletal manifestations are prominent in pediatric SCURVY. Multiple subperiosteal hematomas are an important indicator for diagnosis.

## 1. Introduction

Scurvy, a disease of vitamin C deficiency, has been increasingly reported in recent years [1]. Scurvy occurs in disadvantaged populations with poor intake of fresh fruit or vegetables. However, the incidence of scurvy in the pediatric population is very low. Musculoskeletal manifestations are prominent in pediatric scurvy [1]. 

This is a report of two cases of scurvy which were revealed by subperiosteal hematoma in children with cerebral palsy and developmental delay.

**Case 1**

A 28-month-old boy was admitted to the department of Pediatrics because of musculoskeletal pain, irritability, fever, and pallor. His past medical history was remarkable for psychomotor developmental delay and a seizure disorder managed with long-term carbamazepine and valproic acid administration. He presented with a three-week history of increasing bilateral knee pain, and there was no history of trauma, but bleeding of the gums and pallor were reported.

The patient had a history of poor oral dietary intake he existed on a diet of milk products, and there was a lack of fruits and vegetables. On physical examination, the patient's weight was below the third percentile for his age group; he was febrile (39°C) and had a convergent strabismus; an intense pallor was noted without hepatosplenomegaly or lymphadenopathy. Musculoskeletal examination revealed: swelling and tenderness of the distal ends of the right and the left elbows; swelling and tenderness of the proximal ends of the right and left legs; and bilateral knee effusions. His thighs and legs were swollen and very tender but not hot, and the color of the skin was normal. A pseudoparalysis of the lower limbs was noted and the boy refused to walk. No other remarkable findings were found.

The diagnosis of osteoarthritis or osteomyelitis was suspected, and intravenous antibiotic therapy was started.

The laboratory data results were as follows: white blood cell count: 13900/*μ*L, platelet count: 368.000/*μ*L, and a microcytic hypochromic aregenerative anemia (hemoglobin of 7,5 g/dL). The C-reactive-protein level was high (175 mg/dL) with normal renal function, normal coagulation panel, and normal liver and muscle enzymes.

Rheumatoid factor and antinuclear antibodies were negative. An iron deficiency was confirmed by the low level of iron 9,7 micro-mole/l. However copper (21,8 micro-mole/l) and zinc (16,2 micromole/l) levels were normal. After transfusion, a hemoglobin rate achieved 10,6 g/dL.

**Figure 1:**
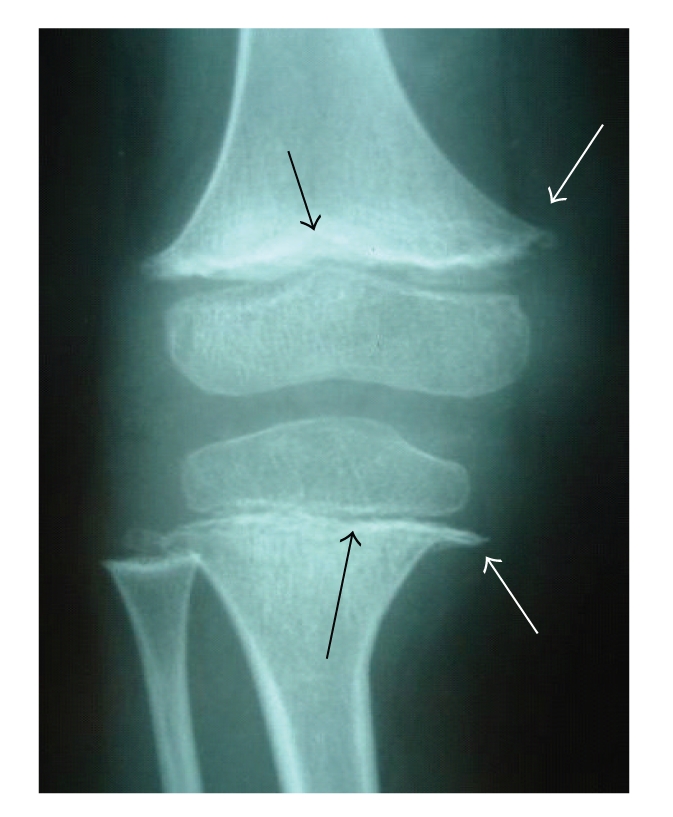
Anterior-posterior radiograph of the knee showing osteopenia, a thickened white line at the metaphysis of both femurs and tibiae (black arrow) and a small beak-like excrescences at the metaphysic of femur and tibi (white arrows).

**Figure 2:**
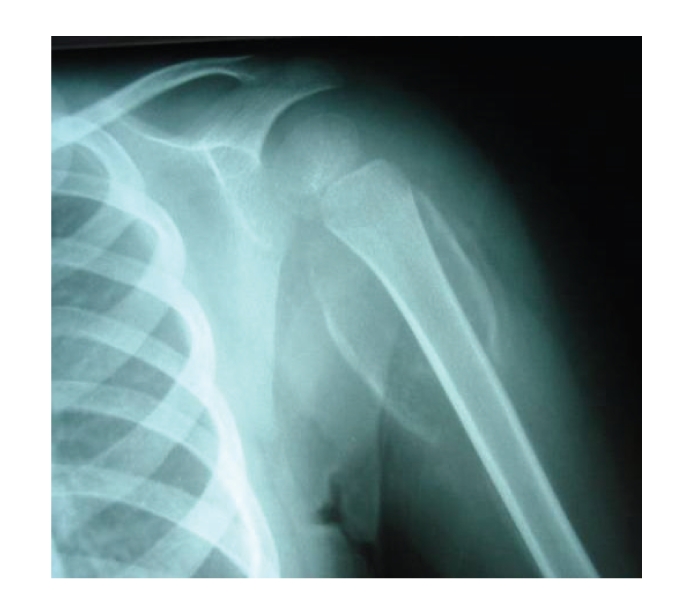
Anterior-posterior radiograph of the left shoulder showing osteopenia and detachment of the periosteum of the humeri.

The radiograph of the knee (Figure 1) showed: osteopenia; an irregular thickened white line at the metaphysis of both femurs and tibiae; a zone of rarefaction under the metaphysis; small beak-like excrescences at the metaphysis of both femurs and tibiae; an increased density outlining the epiphyses; swelling of the soft tissues of the thighs and legs. The radiograph of the shoulders showed detachment of periosteum in humeri (Figure 2).

Ultrasound of the knees showed detachment of the periosteum in the literal-internal face of both femurs and tibias; bilateral effusions of both knees but more pronounced with the left; and infiltration and denseness of the soft tissues.

The diagnosis of subperiosteal fluid collection was evoked, and a bone narrow aspiration of the femur was performed revealing the presence of blood.

A total body computed tomography scan was performed showing the presence of multiple subperiosteal hematoma in the posterior faces and lateral faces of the distal ends of both femurs; subperiosteal hematoma of the upper ends of both tibiae and fibulae; periosteal bone was seen along the shafts of the long bones; osteopenia with irregular contours of the epiphysis; subperiosteal hematoma was also seen in both humeri.

Bone scintigraphy was performed, showing at the tissue phase: increased uptake of the whole right femur and on the low portion of the left femur; at the skeletal phase: symmetric and bilateral high periosteal linear uptake of 99 mTc MDM on the humeri; femur and tibia with important enlargement of the diaphysis.

Because of the predilection of the abnormalities to the metaphyseal equivalents where bone growth normally takes place, we considered metabolic disorders, and in particular scurvy was suspected.

Vitamin C analysis was performed in Pasteur Cerba Laboratory in France; serum ascorbic acid concentration was found to be extremely low, under three micro-mole/l (reference range: 26, 1 to 84, 6 micro-mole/l).

The diagnosis of scurvy was confirmed, and the child was treated with 500 mg of vitamin C daily. His mother was educated about dietary modification. Two weeks after vitamin C administration, the child's pain and the general condition of the patient improved.

**Case 2**

A five-year-old boy presented with pain and swelling of the left lower limb, intense pallor, and mild fever for one month. There was no history of trauma. The patient was diagnosed with cerebral palsy, severe developmental delay, and seizure disorder managed with valproic-acid and vigabatrin administration.

The patient had a history of poor oral intake, and his diet consisted only of milk products.

Upon physical examination, the patient's weight was below the third percentile for his age group; he also had pallor and was irritable. The child was nonambulant with severe spastic quadriplegia. Musculoskeletal examination showed a swelling of the proximal end of the left lower limb without erythema. The child had a decreased range of motion of his knees without joint effusion. No other remarkable findings were found.

The laboratory data results were as follows: white blood cell count: 4400/*μ*L, platelets count: 275000/*μ*L, and microcytic hypochromic aregenerative anemia (hemoglobin of 5 g/dL). An iron deficit was confirmed by the low level of iron 6,6 micro-mole/l (reference range 12 to 27 micro-mole/l). Coagulogram, C-Reactive-Protein, calcium, phosphate, and alkaline phosphatase levels were normal. After transfusion, hemoglobin rate achieved 9,1 g/dL.

**Figure 3:**
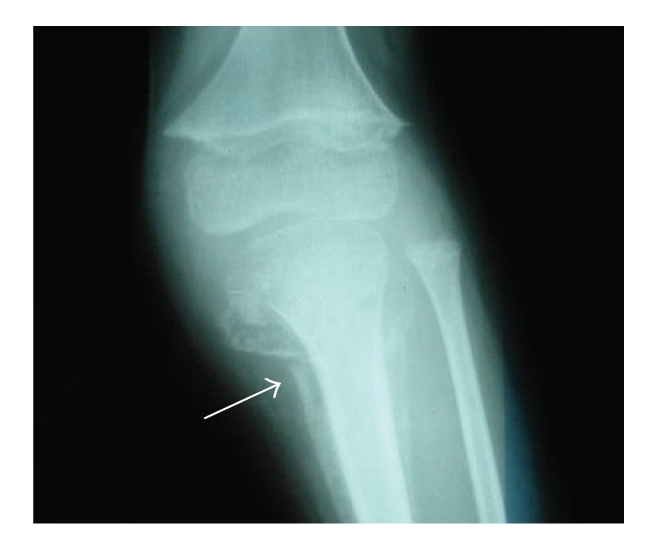
Radiograph of knee showing: an irregular thickened white line at the metaphysis of the femur, a small beak-like excrescences at the metaphysis of the femur, and a detachment of the periosteum of the tibia.

The radiograph of both knees (Figure 3) showed: osteopenia, an irregular thickened white line at the metaphysis of femur, a zone of rarefaction under the metaphysis, small beak-like excrescences at the metaphysis of femur, and detachment of periosteum of the tibia.

Ultrasound of the left knee showed infiltration and denseness of the soft tissues with detachment of the periosteum in the lateral-internal face of the left tibia. Hence the diagnosis of subperiosteal hematoma associated with a hematoma of soft tissues was suggested.

Bone scintigraphy was performed, showing an increased uptake on the whole left tibia and on the left knee.

The study of the vitamin C level in the blood plasma was performed in Pasteur Cerba laboratory in France. Serum ascorbic acid level was found to be extremely low, under three micro-mole/l (reference range: 26,1 to 84,6 micro-mole/l).

The diagnosis of scurvy was confirmed, and the child was treated with 500 mg of vitamin C daily. After vitamin C administration, the child's pain quickly improved.

## 2. Discussion

Scurvy is less common in the pediatric population, but case reports still appear [1–3]. A review of the literature by Noble et al. reveals twenty three case reports of scurvy in children with behaviourally restricted diets including autistic children, mentaly retarded children, and children with cerebral palsy [4].

Musculoskeletal manifestations are present in 80% of patients with scurvy and are prominent in pediatric population [3, 5]. Musculoskeletal manifestations include subperiosteal hemorrhages leading to bone pain and musculoskeletal complaints such as limb pain, limping, swelling over long bones, and progressive leg weakness and fractures [6]. Dermatological manifestations include petechiae, ecchymoses, hyperkeratosis, and perifollicular hemorrhage [3, 7]. Oral symptoms include gingival disease characterized by swelling, ecchymoses, bleeding gums, and loosening of teeth [3, 6, 8]. Systemic symptoms of scurvy in children include lassitude and fatigue, failure to gain weight, loss of appetite, and irritability [6]. In addition to these symptoms, deficiency of ascorbic acid may lead to an hypochromic microcytic anemia because of decreased absorption of iron, bleeding, and dietary deficiencies [3, 6]. Our two cases had severe anemia and received transfusions of packed red cells.

The radiographic findings of infantile scurvy are multiple [9, 10]: the most common is osteopenia but this sign is non specific; more specific signs are less common: a transverse metaphyseal line of increased density called white line or Frankel sign; a transverse metaphyseal bands of decreased density next to the Frankel sign called scurvy lines; osteoporosis of the epiphysis which is surrounded by a white line of calcification called ring sign or Wimberger; lateral metaphyseal excrescences of the beaks secondary to infarction subperiosteal hemorrhages irregular calcification and widening of the costochondral rib junction; and epiphyseal separations. These radiographic findings only become manifest after three to six months of nutritional vitamin C deficiency [9].

The scintigraphic findings in scurvy are as follows: in the early stage there is a generalized increased uptake along the shaft, of the femur without widening of the shaft and when the hematoma becomes organized and calcified, there is a markedly increased club shaped uptake [11]. 

Computed tomography shows osteopenia and joint effusion and reveals the presence of multiple subperiosteal hematomas along the shaft of the femur, tibia, and humeri.

 MRI shows heterogeneous signal intensities along nearly the entire femoral shaft on both T1 and T2 weighted images, and a large collection of subperiosteal fluid with rim enhancement and surrounding soft tissue edema was enhanced. The followup MRI showed a much more notable increase in the amount of subperiosteal hematoma on both femoral shafts; hence the recurrent subperiosteal hematoma was an important clue for the diagnosis of scurvy [9].

Anemia, low serum cholesterol, and albumin levels are found in most patients with scurvy. A low vitamin C concentration in the plasma is specific for the diagnosis of scurvy; however, the result is more heavily dependent on the recent ascorbic acid intake than on the body pool [3]. A serum concentration of vitamin C (<11 micro-mole/l) suggests scurvy.

The best evidence of the presence of scurvy is the resolution of the manifestations of the disease after ascorbic acid treatment [3]. Weinstein et al. [3] recommend oral doses of 100 to 300 mg of vitamin C daily until body stores are replenished per serum levels. Daily fruit and vegetable intakes should include a good source of vitamin C such as citrus, berries, cruciferous vegetables, or peppers. Once a regimen of vitamin C is begun, improvement of symptoms usually begins in 24 hours, with pain diminishing in two to four days, and gingival lesions recovering in two to three weeks [6]. With vitamin C supplementation, metaphyseal abnormalities of scurvy will completely resolve [9]. The large shells of periosteal bone are common radiographic findings particularly during the healing phase of disease [12]. 

Various factors contribute to nutritional deficiencies in non ambulant children with severe spastic cerebral palsy like poor intake, oral motor dysfunction, feeding problems, and use of antiepileptic drugs [13]. 

In conclusion, scurvy is rare in children. Musculoskeletal manifestations are prominent in pediatric scurvy. The diagnosis of scurvy is made by clinical and radiographic findings and may be supported by reduced concentration of vitamin C in the serum. In children with eating difficulties, it is essential to prevent scurvy by systematic dietary supplementation of vitamin C.
